# Nutrition and Health Disparities: The Role of Dairy in Improving Minority Health Outcomes

**DOI:** 10.3390/ijerph13010028

**Published:** 2015-12-22

**Authors:** Constance Brown-Riggs

**Affiliations:** Nutrition Consultant CBR Nutrition Enterprises, Massapequa 11758, NY, USA; cbr5274@aol.com

**Keywords:** health disparities, 2015 dietary guidelines, WIC, yogurt, National Medical Association, MyPlate, African American, Hispanic American, National Hispanic Medical Association

## Abstract

Consuming a balanced diet, such as the food groups represented on MyPlate, is key to improving health disparities. Despite the best of intentions, however, the dietary guidelines can be culturally challenging, particularly when it comes to dairy consumption. Many African and Hispanic Americans avoid milk and dairy products—key contributors of three shortfall nutrients (calcium, potassium and vitamin D)—because many people in these populations believe they are lactose intolerant. However, avoiding dairy can have significant health effects. An emerging body of evidence suggests that yogurt and other dairy products may help support reduced risk of heart disease, hypertension, obesity, and type 2 diabetes—conditions that disproportionately impact people of color. For this reason, the National Medical Association and the National Hispanic Medical Association issued a joint consensus statement recommending African Americans consume three to four servings of low-fat dairy every day. Cultured dairy products could play an important role in addressing these recommendations. Because of the presence of lactase-producing cultures, yogurt is often a more easily digestible alternative to milk, and thus more palatable to people who experience symptoms of lactose intolerance. This was a key factor cited in the final rule to include yogurt in the Special Supplemental Nutrition Program for Women, Infants, and Children.

## 1. Introduction

The 2010 Dietary Guidelines for Americans (DGA) identified nine nutrients—vitamins A, D, E, and C; folate; calcium; magnesium; fiber; and potassium—as “short-fall nutrients”: those that are under-consumed by a significant portion of Americans. Because of the association in the scientific literature with adverse health outcomes, four of these—calcium, vitamin D, fiber, and potassium—are classified as “nutrients of public health concern”, *i.e.*, nutrients the overconsumption of which may cause health risks in specific populations or populations at large. The DGA also considers sodium and saturated fats nutrients of public health concern because these food components are consumed in excess [1]. 

The question is: What can be done to address the under-consumption of these important nutrients—particularly in a way that also addresses the related health concerns? Dairy foods such as milk, cheese, and yogurt can be key in that they deliver many nutrients important for good health, including three of the nutrients of public health concern—calcium, potassium, and vitamin D. In fact, according to the 2010 DGA, three servings of vitamin D fortified low-fat and nonfat milk and milk products would provide 70% of the calcium and vitamin D, and 30% of the potassium in the diet [1]. Health authorities such as the American Diabetes Association (ADA), the American Heart Association (AHA), the National Medical Association (NMA), and the National Hispanic Medical Association (NHMA) all recommend three servings of low-fat dairy per day as a means of closing the nutrient intake gap [2,3,4]. 

It is significant, but not surprising that the latter two organizations, the NMA and NHMA, have addressed this public health concern. As stated in the 2010 DGA evidence rating, moderate evidence shows that the intake of milk and milk products is associated with a reduced risk of cardiovascular disease, type 2 diabetes, and lower blood pressure in adults—disease states affecting African Americans (AA) and Hispanic Americans (HA) at disproportionate rates [1]. The 2015 Dietary Guidelines Advisory Committee (DGAC) reaffirmed this association in their scientific report [5]. This evidence makes a strong case for the inclusion of dairy in the diets of AA and HA. It is understood that health disparities may exist among all racial and ethnic minority groups, however, this article will focus on dairy's role in improving AA and HA health outcomes, and strategies for increasing dairy consumption among these populations. 

## 2. Key Factors

### 2.1. Minority Health Disparities

Research shows that the rates of obesity, diabetes and heart disease are higher in AA and HA populations than in white populations (Table 1). From 2011 to 2012, the greatest prevalence of obesity was among AA adults followed by HA adults [6]. In 2011, the prevalence of diabetes among AA adults was nearly twice as great as the prevalence among white adults [7]. Likewise, the prevalence of heart disease was greatest in AA compared with HA and white adults. African Americans not only experience higher prevalence rates of these health conditions, but higher mortality rates as well. For example, in 2013 death rates from heart disease were greatest among AA compared with other racial populations and AA were twice as likely to die from diabetes complications [8,9]. 

**Table 1:** Prevalence of chronic disease by ethnicity [6,7,8,9].

	Obesity	Diabetes	Heart Disease
Non-Hispanic Black	47.8%	12.7%	7.0%
Hispanic	42.5%	12.1%	5.9%
Non-Hispanic White	32.6%	7.3%	6.3%

### 2.2. Dairy Intake and Chronic Diseases

There is evidence that dairy foods and the important nutrients they contain—namely, calcium, vitamin D and potassium—are linked to reduced risk of heart disease; type 2 diabetes; and metabolic syndrome, which is responsible for obesity and diabetes [3]. These are all conditions that AA and HA populations experience in disproportion—a fact that is perhaps related to the fact that minority populations often have lower intake of key nutrients of concern: calcium, potassium and vitamin D (Table 2). African Americans fall behind HA and the white populations, consuming only 83% of the daily recommended intake of calcium, 27% of vitamin D, and 50% of the potassium (Table 3) [10].

**Table 2:** Nutrient intake from food: mean amounts consumed per individual, by race/ethnicity, age 19–50 [10,11].

	Calcium (RDA)	Vitamin D (RDA)	Potassium (AI)
	**1000 mg/d**	**15 mcg/d**	**4700 mg/d**
Non-Hispanic White	1070	5.4	2868
Mexican American	975	4.9	2758
All Hispanic	969	4.8	2711
Non-Hispanic Black	828	4.1	2364

**Table 3:** Percent of daily recommended intake derived from calculating actual intake divided by Recommended Dietary Allowance (RDA) or Adequate Intake (AI), age 19–50 [10,11].

	Calcium (RDA)	Vitamin D (RDA)	Potassium (AI)
	**1000 mg/d**	**15 mcg/d**	**4700 mg/d**
Non-Hispanic White	107%	36%	61%
Hispanic	97%	32%	58%
Non-Hispanic Black	83%	27%	50%

The connection between the shortfall in nutrient consumption and increased risk of these adverse health conditions can be explained by looking at the ways that these nutrients—or deficiency thereof—impact hypertension, diabetes, and obesity. For example, insufficient potassium is associated with hypertension, but consuming dietary potassium can lower hypertension by blunting the adverse effects of sodium on blood pressure. Evidence suggests that AA and individuals with hypertension especially benefit from increasing intake of potassium [1]. Calcium plays a critical role in nerve transmission, muscle contraction, and the constriction and dilation of blood vessels [1]. Adequate calcium intake may be particularly critical for AA and HA since research among these groups revealed higher diagnoses rates of diabetes and hypertension [1]. A calcium-rich diet (1000 mg or more daily) has been shown to decrease blood pressure and inhibit lipogenesis in the fat tissue, thus additionally improving cardiovascular risk [12]. But to help absorb calcium, Vitamin D is needed and African Americans may be at a higher risk for vitamin D deficiency due to dark pigmentation which blocks absorption of vitamin D from the sun [4]. In the United States, especially in colder zones where people get less daily sun exposure, most dietary vitamin D in the diet comes from fortified foods, especially milk and yogurt [1]. 

From this evidence, it is clear that fortified dairy foods can play an important role in addressing some of the health conditions that hit Black communities the hardest. 

#### 2.2.1. Obesity

The relationship between dairy and AA/HA health is more complicated when it comes to the impact of dairy on obesity. Dairy foods, particularly full-fat varieties, are often associated with excessive weight gain, which in turn contributes to diabetes, heart disease, and other conditions. However a 2013 prospective population-based cohort study of over 1700 men, aged 40–60 years, concluded a high intake of dairy fat was associated with a lower risk of central obesity (OR 0.52, 95% CI 0.33–0.83) and a low dairy fat intake was associated with a higher risk of central obesity (OR 1.53, 95% CI 1.05–2.24). High consumption of dairy fat was defined as butter, full fat milk and intake of whipping cream daily or several times a week. Low consumption of dairy fat was defined as no butter, low fat milk (1.5% fat or less), and seldom or never ingesting cream [12].

In one prospective study, researchers examined three separate cohorts of more than 120,000 US women and men followed every four years for twenty years. Evidence showed that consumption of yogurt, fruits, vegetables, and whole grains was associated with less weight gain over time, with yogurt having the greatest impact. Weight change was inversely associated as follows: vegetables (−0.22 lb), whole grains (−0.37 lb), fruits (−0.49 lb), nuts (−0.57 lb), and yogurt (−0.82 lb) (*p* ≤ 0.005 for each comparison) [13].

#### 2.2.2. Heart Disease

Hypertension is another area of critical health concern for minority communities. High-blood pressure increases the risk for cardiovascular disease, including heart attack and stroke [14] and uncontrolled hypertension is higher among AA and HA than whites [15]. It is one of the top health concerns for African Americans.

One of the interventions recommended to patients diagnosed with hypertension is the Dietary Approaches to Stop Hypertension (DASH) eating plan. The DASH diet recommends lowering sodium intake; eating foods high in blood-pressure lowering nutrients such as calcium, potassium, and magnesium; and consuming fat-free and low-fat milk and milk products. Clinical trials show that the DASH eating plan is not only effective in lowering blood pressure, benefit of DASH was more pronounced in African Americans. Blood pressure was reduced by 6.9 mmHg systolic and 3.7 mm Hg diastolic in African Americans compared to 3.3 mm Hg and 2.4 mm Hg, respectively, in whites [16].

In a 2013 prospective study of more than 33,000 women in Sweden, researchers examined the association between total, as well as specific, dairy food (milk, cultured milk/yogurt, cheese, cream, crème fraiche, and butter) intakes and the incidence of myocardial infarction (MI). Evidence showed an inverse association between total dairy food intake and risk of MI. No difference was observed between specific dairy foods, nor between low-fat and full-fat dairy foods as it relates to risk of MI [17]. 

A 2013 literature review published in *Nutrition Reviews* was conducted to determine whether or not there was sufficient evidence to elucidate or dismiss an association between dairy foods and blood-pressure maintenance. The authors concluded that the preponderance of evidence indicates low-fat, non-fat, and full-fat dairy foods are beneficially associated with lower blood pressure [18].

In one 2013 cross-sectional study of adults in the Framingham Heart Study Offspring and Third Generation cohorts, yogurt consumers had higher potassium intakes, lower levels of circulating triglycerides and glucose, and lower systolic blood pressure and insulin resistance when compared with non-yogurt consumers [19]. The study authors suggest the metabolic changes may be due in part to an association between yogurt consumption and BMI. 

#### 2.2.3. Diabetes

Evidence suggests that dairy consumption may be associated with a reduced risk of type 2 diabetes. A meta-analysis of seven cohort studies showed an overall positive role of dairy consumption on the risk of type 2 diabetes, with authors reporting a 14% reduced risk of the disease associated with highest (>3 serving/day) *versus* lowest (<1 serving/day) dairy food intake [20]. A subgroup analysis suggested that low-fat milk and yogurt consumption were most strongly associated with diabetes risk reduction when compared to high-fat dairy. One serving of dairy per day was found to reduce the risk of type 2 diabetes by 5%. Adding one additional serving of low-fat dairy reduced risk by 10% [20]. In contrast, low-level dairy consumption, for example in the case of self-perceived lactose intolerance, results in lower intakes of calcium and other nutrients and is associated not only with diabetes but hypertension as well [21].

An August 2013 meta-analysis analyzed 17 cohort studies with data from more than 370,000 men and women to determine the relationship between dairy intake and diabetes risk. Evidence suggests a significant inverse association between intake of dairy products—including low-fat dairy products and cheese—and type 2 diabetes risk. Moreover, a dose-response analysis showed that for every 400 g of total dairy per day, type 2 diabetes risk was reduced by 7% [22]. 

The type of dairy may also be beneficial for the prevention of type 2 diabetes. A 2014 prospective study found that higher consumption of low-fat fermented dairy products compared to high fat fermented diary was associated with a decreased risk of type 2 diabetes. This response was largely driven by yogurt consumption. Fermented dairy products in the study included yogurt, cheese, sour cream, and crème fraîche [23].

### 2.3. Lactose Intolerance

The true prevalence of lactose intolerance is not known as the condition is fairly difficult to diagnose with accuracy. However, perceived lactose intolerance is a major health concern. Research shows that currently 20.1% of AA and 8.8% of HA, compared to 7.8% of non-Hispanic whites, consider themselves to be lactose intolerant [21,24]. Even in cases where there is no definitive diagnosis, patients may assume or believe they are lactose intolerant and modify their diet accordingly—often eliminating a great deal of their dairy intake and, thus, the nutritional benefits that come from eating dairy foods.

In order to get to the recommended daily values of calcium, vitamin D, and potassium, public health authorities recommend three servings of dairy every day [1]. For African American and HA adults, age 20 years and over, dairy intake is less than recommended: African Americans 1.19 (SE 0.005) servings daily, HA consume 1.49 (SE 0.063) servings daily and whites consume 1.89 (SE 0.055) servings daily [10]. For those who perceive themselves to be lactose intolerant, milk avoidance is a major obstacle in obtaining adequate calcium and vitamin D from the diet, and it has been shown that avoiding dairy may lead to shortfalls in essential nutrients [4,24]. Because of this nutritional shortfall, the National Institutes of Health identified self-restriction of dairy foods associated with self-diagnosis of lactose intolerance as a public health problem [25].

### 2.4. Health Benefits of Yogurt

Lactose intolerant individuals—including those whose intolerance is perceived—can meet their dairy requirement and obtain critical nutrients of concern by consuming yogurt [26]. Many people who avoid dairy products because they are lactose intolerant may find that the live and active cultures in yogurt can improve lactose digestion. Yogurt contains less lactose per serving than milk, making it a more digestible alternative (Table 4) [27].

**Table 4:** Lactose in common dairy food [27].

Food	Serving	Amount Lactose
Whole, 2%, 1%, Skim Milk	1 cup	12 g
Chocolate, milk, reduced fat	1 cup	10 g
Lactaid	1 cup	0 g
Cottage Cheese, low-fat, 2% milk fat	½ cup	3 g
Cheddar Cheese, sharp	1 oz.	<0.1 g
Swiss Cheese	1 oz.	<0.1 g
Mozzarella	1 oz.	<0.1 g
Queso Fresco	1 oz.	<0.1 g
American Cheese, pasteurized processed	1 oz.	1 g
Yogurt, plain, whole milk	6 oz.	8 g
Yogurt, Greek, plain, nonfat	6 oz.	4 g

Yogurt is also rich in the nutrients needed to address many adverse health conditions. For example, a single 8-ounce serving of yogurt provides 6%–14% of the recommended daily intake for potassium [28]. Many fat-free and low-fat yogurts provide approximately 25% more potassium than an equal 8-ounce serving of milk [27]. Overall, yogurt consumers have a higher potassium intake, and are less likely to have inadequate intakes of calcium and magnesium [19]. Increasing the proportionate intake of fat-free and low-fat yogurt and milk would not only increase potassium levels, but also increase levels of magnesium, vitamin A, D, and choline in the USDA Food Patterns, and potentially decrease amounts of sodium, cholesterol, and saturated fat [28]. 

Studies have shown that regular yogurt consumption is associated with a healthy weight, decreased waist circumference, healthy levels of circulating glucose within the normal range, and decreased blood pressure [13,19]. The American Diabetes Association recommends plain, nonfat Greek yogurt as a good choice for people with diabetes [29].

### 2.5. WIC Program Expansion

The Special Supplemental Nutrition Program for Women, Infants and Children (WIC) is a federal program that provides nutrition education and vouchers for nutritious foods to low-income pregnant, breastfeeding, and non-breastfeeding postpartum women, and to infants and children up to age 5 who are found to be at nutritional risk. WIC food packages and nutrition education are the chief means by which WIC affects the dietary quality and habits of participants. Since its inception in 1974, WIC has earned the reputation of being one of the most successful federally funded nutrition programs in the United States [30]. 

In 2007, the WIC food packages were revised to more closely align with the latest DGAs, and provide WIC participants with a wider variety of food. The changes also gave WIC State agencies greater flexibility in prescribing food packages to accommodate participants with cultural food preferences and address regional food trends and traditions. The revisions largely reflected recommendations made by the Institute of Medicine (IOM) of the National Academies in its report “WIC Food Packages: Time for a Change.” To address the nutritional needs of individuals who avoid milk due to cultural food preferences or lactose maldigestion, the IOM recommended yogurt be added as a substitute for part of the milk allowance. Heretofore, yogurt had not been on the list of foods that WIC covered [30]. 

In order to address this recommendation, the USDA asked states for assistance in exploring how yogurt could be provided and incorporated into the diet [30]. They wanted to determine acceptability and cost implications. In response, the California WIC Program conducted a randomized, controlled intervention pilot study to examine the impact of providing yogurt to women enrolled in WIC. The objective of the study was to document changes in women’s preferences for yogurt, perceived barriers to yogurt consumption, and the number of dairy servings consumed overall. The study allowed participants to replace part of the WIC milk allowance with yogurt. It found that over 86% of the 511 women in the study wanted to do so. Among these, 62% reported preferring yogurt to milk. The majority (89%) of women in the study redeemed the yogurt coupons [31]. 

The study concluded that, when the WIC program allowed it, yogurt was a popular substitute for milk that tackled at least two concerns related to dairy consumption: It addressed the dietary needs, and concerns of WIC participants who were either lactose intolerant or irregular milk drinkers. It also addressed a cost barrier to consuming yogurt that study participants had noted. Yogurt was perceived as being more expensive than other dairy products. Removing this barrier could address income-related disparities that affect diet quality [31]. 

The final changes to the modified WIC food package, effective April 2015, reflect the results of the California research. In response to the recommendations of the IOM, the WIC food package now includes yogurt as a partial substitute for milk [30]. One quart of yogurt may be substituted for one quart of milk, with no more than one quart of yogurt per month. 

## 3. Recommendations

The evidence-based Dietary Guidelines are used by the U.S. government as the basis for its food assistance programs, nutrition-education efforts, and decisions about national food-related health objectives. Urging people to consume a balanced diet—one that includes the food groups represented on MyPlate—is key to improving diet-related health disparities. But despite best intentions, the guidelines can be culturally challenging. For example, people with perceived lactose intolerance—a group that includes a disproportionate number of African Americans—can find it difficult to follow the guidelines for dairy intake. 

In their joint 2013 consensus statement on lactose intolerance, The National Medical Association and the National Hispanic Medical Association recommended that healthcare providers encourage patients to keep dairy foods in the diet, even if they are lactose intolerant. The NMA and NHMA encouraged providers to help patients employ strategies to help them achieve the recommended dairy food intake levels [4]. One such strategy is to replace milk servings with cultured yogurt. Nutrition programming should include research-based information on yogurt’s nutritional quality, purchasing and handling guidelines, and recipes for meals and snacks. Such nutrition programming and communication would help ensure that minorities are provided with options for meeting the recommended nutrient and food group intake. 

Yogurt provides valuable nutrition contributions and should be considered as the 2015 Dietary Guidelines are implemented. Yogurt should also be included in the revised WIC packages, and education messages should be developed that include the benefits of including yogurt in the diet. Recommendations should be culturally relevant and practical to ensure best outcomes.

## 4. Conclusions 

Dairy is an important dietary component, contributing nutrients that address conditions for which minorities are measurably at risk. Because AA and HA populations tend to have less-than-optimal intakes of key nutrients, they could benefit from the inclusion of recommended amounts of dairy in their diet. There are also health-disparity implications that come from increasing dairy intake, given the documented association between dairy consumption and lowered risk of obesity, type 2-diabetes, and hypertension. Among dairy products, yogurt has all the benefits of dairy, plus the benefit of being more easily digestible among people who perceive that they have a problem with lactose intolerance. When given the option, WIC clients demonstrated their preference for yogurt by using their vouchers to purchase this product. As a result, WIC has included yogurt to the list of other healthy foods that are available to WIC recipients. All the benefits and uses of yogurt should be promoted in the implementation phase of the 2015 DGA.
